# Structural Elucidation, Modification, and Structure-Activity Relationship of Polysaccharides in Chinese Herbs: A Review

**DOI:** 10.3389/fnut.2022.908175

**Published:** 2022-05-20

**Authors:** Bei Wang, Lingling Yan, Shuchen Guo, Ling Wen, Mengli Yu, Liang Feng, Xiaobin Jia

**Affiliations:** State Key Laboratory of Natural Medicines, School of Traditional Chinese Pharmacy, China Pharmaceutical University, Nanjing, China

**Keywords:** Chinese herbal polysaccharides, structure-activity relationship, structural elucidation, modification, bioactivity

## Abstract

Chinese herbal polysaccharides (CHPs) are natural polymers composed of monosaccharides, which are widely found in Chinese herbs and work as one of the important active ingredients. Its biological activity is attributed to its complex chemical structure with diverse spatial conformations. However, the structural elucidation is the foundation but a bottleneck problem because the majority of CHPs are heteropolysaccharides with more complex structures. Similarly, the studies on the relationship between structure and function of CHPs are even more scarce. Therefore, this review summarizes the structure-activity relationship of CHPs. Meanwhile, we reviewed the structural elucidation strategies and some new progress especially in the advanced structural analysis methods. The characteristics and applicable scopes of various methods are compared to provide reference for selecting the most efficient method and developing new hyphenated techniques. Additionally, the principle structural modification methods of CHPs and their effects on activity are summarized. The shortcomings, potential breakthroughs, and developing directions of the study of CHPs are discussed. We hope to provide a reference for further research and promote the application of CHPs.

## Introduction

The polysaccharides derived from Chinese herbs are mostly heteropolysaccharides which consist of different kinds of monosaccharides. Modern pharmacological studies reported that CHPs had functions such as anti-tumor (1), immunologic enhancement (2), intestinal microenvironment regulation (3), and anti-oxidation (4). As a drug, it is related to the occurrence and treatment of a variety of diseases and is favored on clinical settings. CHP preparations such as *Astragalus* polysaccharide injection, *Ginseng* polysaccharide injection, and *Poria cocos* polysaccharide oral liquid have been widely applied for clinical use.

Previous research focused on small molecules in Chinese herbs, while ignoring the research on macromolecular polysaccharides, resulting in a waste of resources. The complexity and instability of polysaccharides' structure make the process of revealing its mechanism and the development of products or drugs more complicated. As a result, it will impede the development of new polysaccharide drugs and reduce the application scope of polysaccharides. To play the better role of CHPs, the structure-activity relationship of polysaccharides must be clarified.

The primary structure and chain conformation of polysaccharides are closely related to the construction of various functions. However, due to the intricate structure and imperfection of technical support, current methods cannot fully clarify the exact structure of CHPs, especially the three-dimensional structure. There are fewer reports on the structure-activity relationship. In addition, some studies have shown that not all polysaccharides can express their ideal bio-activities (5). Nevertheless, the above problem is effectively solved by modifying its structure. Biological activity of polysaccharides after modification can be enhanced greatly and even new biological activity can be produced (6, 7). Structural modification can improve the physicochemical properties and activities of polysaccharides, which promotes the exploration of the structure-activity relationship of polysaccharides. Therefore, we summarized the structural elucidation, modification, and structure-activity relationship of CHPs, so as to provide theoretical reference and technical support for the development and utilization of CHPs.

## Structure-Activity Relationship

**Figure 1:**
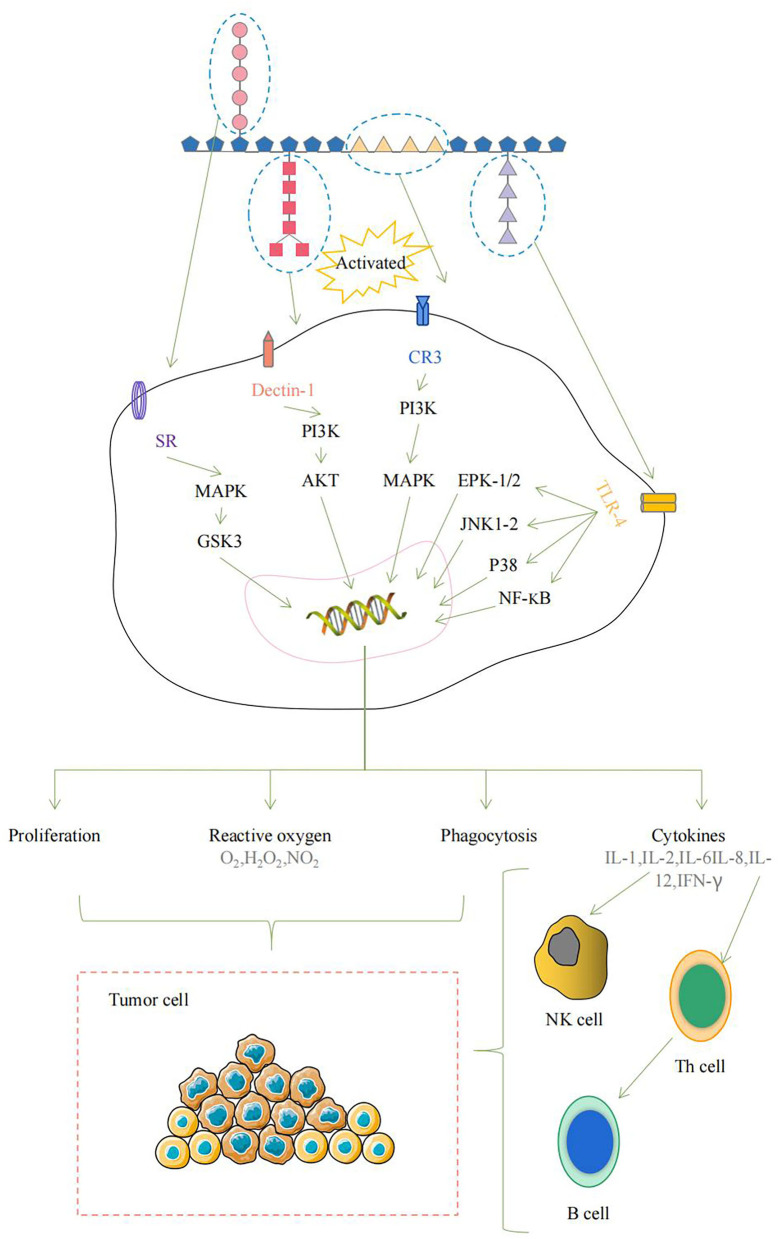
The schematic diagram of the immune system activated by *Marine algae* polysaccharides after interaction of several molecular events.

Various polysaccharides show certain chemical structures. Wherein their biological activities depend on events that occur at the molecular structure level (8). Therefore, exploring the structure-activity relationship of polysaccharides in CHPs is of great significance for the development of new-carbohydrate drugs or pharmaceutical excipients. For example, different structural parts of Marine algae polysaccharides can directly or indirectly interact with the immune system and trigger several signal pathways, which lead to immune system activation (Figure 1) (9).

### Relationship Between Molecular Weight and Activity

The molecular weight of polysaccharides only represents the average distribution within a certain range of relative molecular mass, which is mainly expressed by the mass average molar mass (M_w_) and number-average molecular weight (M_n_). Polydispersity (α) is generally used to describe the molecular weight distribution of polysaccharides, that is, α = M_w_/M_n_.

Firstly, molecular weight is an important feature affecting the therapeutic action of polysaccharides. The functional feature of molecular weight is related to amounts of active groups, and molecular weight indirectly affects the physicochemical properties such as solubility and viscosity, thereby affecting the absorption of polysaccharides *in vivo*. Secondly, the higher or lower molecular weight might reduce the activity of CHPs. It should be noted that a high molecular weight of polysaccharide normally has a large excluded volume that promotes intermolecular interaction of polysaccharide and impedes its uptake. On the contrary, if the relative molecular weight is too low, polysaccharides cannot form active-polymer structures such as triple-helical conformation (10). For example, the polysaccharide with moderate molecular weight from *Dendrobium officinale* showed the strongest inhibitory effect on the cells (11). Polysaccharides with less than 5 kDa or more than 400 kDa showed marginal immunomodulatory activity or lost the activity directly (12). Therefore, the dimension of molecular weight is closely related to biological activities in polysaccharides including antioxidation, lipid-lowering, antiviral, and so on.

Thirdly, the molecular weight shows a certain tendency of efficacy in an appropriate range. The low molecular weight of polysaccharides can play better exerting their antioxidant activity with containing more free hydroxyl groups to accept and eliminate more hydrogen radicals (13). It was found that a polysaccharide with the lower molecular weight obtained from corn whiskers showed the stronger antioxidant activity (14). It is worth noting that CHPs with intestinal barrier protection have higher molecular weight (15), which may be because high molecular weight polysaccharides can maintain the integrity of intestinal barrier structure by forming something similar to the sticky gel (16).

Taken together, polysaccharides in CHPs perform the best only on specific biological activities within the optimal range of relative molecular weight.

### Relationship Between Monosaccharide Composition and Activity

The monosaccharide composition is divided into the type and proportion of monosaccharide, which is closely associated with biological activity. As an illustration, *Angelica sinensis* polysaccharides with radio-protective activity tended to be richer in galacturonic acid, galactose, and arabinose (17). It is generally considered that the monosaccharide composition with more complex shows the better biological activity (18). Two kinds of polysaccharides, RLP-1 and RLP-2, from *Rosa Laevigata* Fructus, had different monosaccharide compositions and activities. RLP-1 consisted of xylose, mannose, and galactose reducing hyperlipidemia of model rats, while RLP-2 only contained glucose having no such activity (19). Some studies demonstrated the monosaccharide composition in polysaccharide with intestinal barrier protection function is galactose, mannose, arabinose, xylose, and rhamnose (16, 20). In addition, the high content of uronic acid showed good antioxidant activity of polysaccharide (21). The mechanism may be the breakage of uronic acid chain caused by free radicals (22). Similarly, polysaccharides with amounts of uronic acid were beneficial to its hepatoprotective activity (18). In addition, the polysaccharides containing mannose and rhamnose were proved to have tumor-inhibitory and antioxidant activities, individually (23, 24). However, the specific rules and mechanisms have not been clearly set forth and need further exploration.

### Relationship Between Glycosidic-Bond Type and Activity

The flexible connection between monosaccharides causes the complex structure of polysaccharides. The glycosidic bond is divided into α-type or β-type because the configuration of the glycosidic bond is determined by the configuration of the hemiacetal (ketone) hydroxyl group. It is generally considered that polysaccharides with β configuration have the higher activity, while most of polysaccharides with α configurations have no biological activity (25, 26). This is due to the existence of α-glucoamylase in the human body, which can hydrolyze α-glycosidic bonds under certain conditions. However, with the depth of research, it was found that α-glucan as vaccine adjuvants had good biocompatibility and biodegradability to maintain the homeostasis of the intestinal environment (27, 28). In addition, α-(1 → 4)-GalpA and α-(1 → 4)-Galp in the main chain of *Ginseng* polysaccharides were essential to biological activities such as anti-tumor (29).

The main glycosidic bond types of polysaccharides with different activities are also different. Most of the glucans with anti-tumor are mainly composed of the β- (1 → 3)-*D*-glucan as the main chain, with the β-(1 → 6)-*D*-glucan randomly as the branched chain (30), while the antitumor effect of glucans composed of the β-(1 → 6)-*D*-glucan as the main chain is much weaker. Some CHPs regulating intestinal flora activity are mostly connected by (1 → 3) glycosidic bond (31). Additionally, CHPs with hypoglycemic effect mostly have (1 → 3), (1 → 4), (1 → 6) glycosidic bonds (32–34).

### Relationship Between Branching Degree and Activity

The degree of branching (DB) of CHPs influences the biological activity by affecting the molecular weight and conformation (35). Generally speaking, the higher the complexity of the branch, the stronger the activity of CHPs (36, 37). Three kinds of polysaccharides with different DBs were obtained from *Ganoderma atrum*. Their antioxidant activity and anti-tumor cell proliferation were positively correlated with DB (38). This may be related to the effect of DB on the binding ability of polysaccharides to specific receptors (39, 40). In recent years, the hyperbranched polysaccharides with highly branched structure have received extensive attention due to their diverse biological activities and applications, which may be because the hyperbranched structure has good water solubility, low viscosity, and high chain end density (41). A hyperbranched polysaccharide from *Cordyceps sinensis* with DB of 43% could stimulate macrophage function, which was thought to attribute to its hyperbranched structure (42). However, it was found that polysaccharide from ginseng with small branching degrees showed better immune-enhancing activity (43). Accordingly, the activity of CHPs is related to DB, and there may be an optimal DB value (44). This may be because too high DB value would lead to the decrease of water solubility, while the DB value is too low, resulting in fewer binding sites (45).

### Relationship Between Chain Conformation and Activity

Compared with the primary structure, advanced structure of polysaccharides is more likely to affect their functions (46, 47). Three-dimensional network structures of polysaccharide molecules are easily formed *via* van der Waals forces, hydrogen bonds, and covalent bonds. Moreover, the inter-chain association of structures is common because of the large degree of freedom and flexibility. Polysaccharides have multiple conformational forms in solutions, including single random coil (48, 49), helix (50), double helix (51), triple helix (52), rod-like structures (53), worm-like (54), semiflexible chains (55), and sphere-like structures (56). At present, the triple-helical structure of polysaccharides is one of the most studied chain conformations with some specific biological activities, especially anti-tumor (57). If triple-helical structure is destroyed, the anti-tumor activity would be decreased. The lentinan had a good antitumor activity with triple-helical stereo-configuration, but when the stereo-configuration was destroyed, the anti-tumor activity disappeared as consequence (58). The antiviral activity could be affected by the change of triple-helical structure of pollen polysaccharides in *Pinus massoniana* (59), which might be related to the chain rigidity and exposure sites of the triple-helical structure. Furthermore, the polysaccharide chain with triple-helical conformation is more rigid and easier to be recognized by the receptor (60). When the polysaccharide with triple-helical structure is linearly distributed, more exposure sites were utilized to strengthen the activity. The research substantiated that the polysaccharide having extended linear conformation showed the better good hypolipidemic activity on four sulfated polysaccharides in sea cucumber (61).

At present, there are few studies about the relationship between the chain conformation and biological activity in polysaccharides, which is one of the key breakthroughs for the advancement of polysaccharides in CHPs.

## Primary Structure Identification

### Determination of Molecular Weight

**Figure 2:**
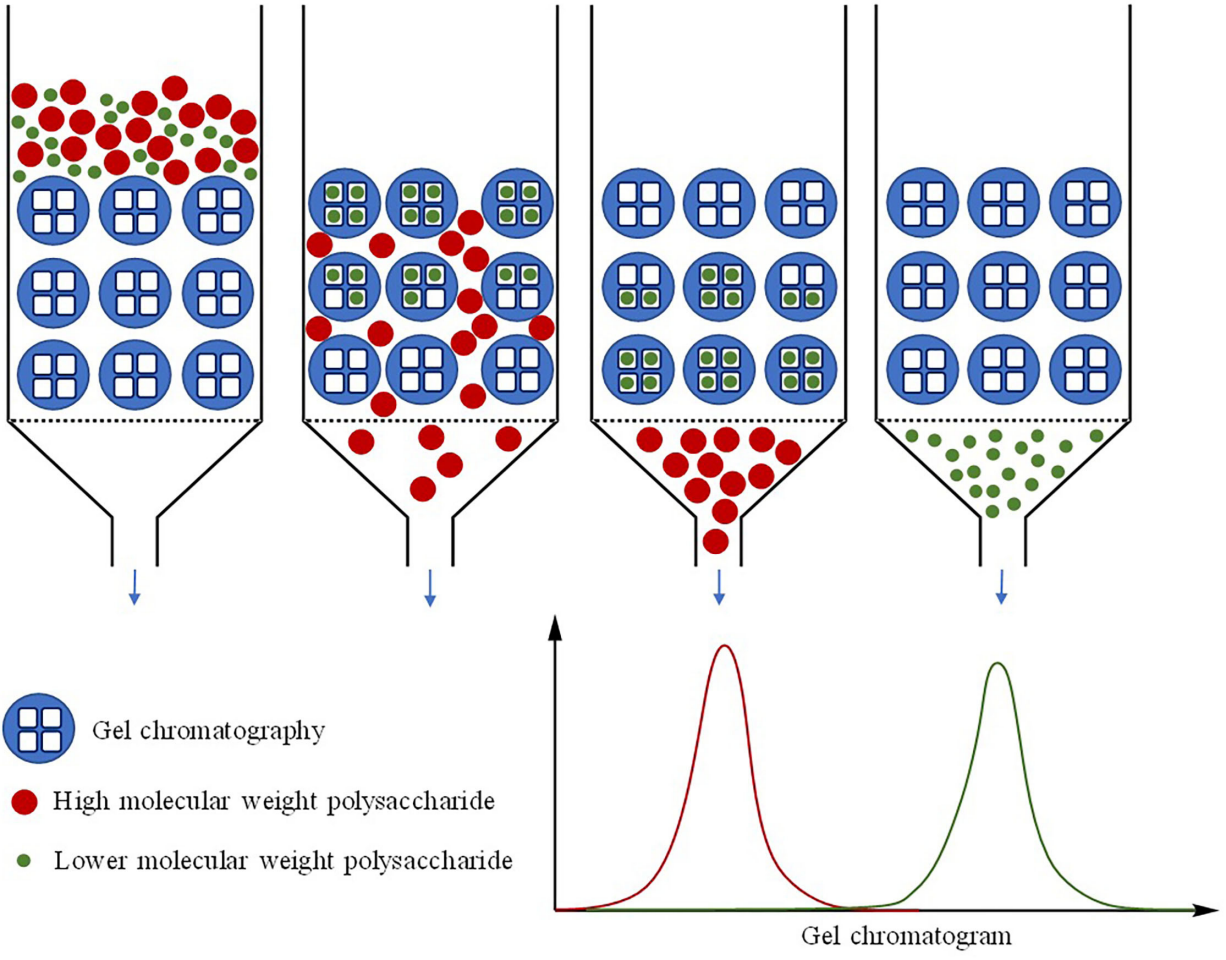
The schematic diagram of mechanism for determination of molecular weight of CHPs by GPC.

In recent years, gel permeation chromatography (GPC) has been widely used in laboratories to calculate the molecular weight of the polysaccharide as well as detect the homogeneity of the polysaccharide (Figure 2) (62, 63). Most of the current studies employ high-performance GPC (HPGPC) or high-performance exclusion chromatography (HPSEC) (64), which is based on the characteristic that polysaccharide with certain molecular weight has a corelated elution retention time (t_R_) on a gel column. Polysaccharides with known molecular weight are used to make a standard curve, and then the molecular weight of the sample can be calculated according to the t_R_ and the standard curve. In this method, if a single and symmetrical peak appears, the component is usually considered to be a homogeneous polysaccharide (65). Differential refraction index detector (RID) in HPGPC was applied to determine the molecular weight of polysaccharides. As long as the refractive index of the detected compound is different from the liquid solvent system, it can be detected. The universal detector evaporative light scattering detector (ELSD) can also be used to determine the molecular weight of polysaccharides (66).

In addition, some hyphenated techniques have been developed to determine the molecular weight of polysaccharides, such as size exclusion chromatography (SEC)/GPC combined with a multi-angle laser light scattering (MALLS) detector (67, 68). A small-angle laser scattering device can be used to determine the molecular weight of macromolecular compounds quickly and accurately, without extrapolating the angle and concentration. The comparison of popular methods on determination molecular weight is shown in Table 1.

**Table 1:** Comparison of determination methods of molecular weight.

	**Method**	**Characteristic**	**Application**	**References**
Traditional techniques	Osmotic pressure method, Viscosimetry, Vapor pressure osmometry, Terminal method, Sugar Electrophoresis, Ultrafiltration	Complicated to operate, inaccurate	Tea polysaccharide (TPS) Mw 2.287 × 10^5^-2.762 × 10^5^ g/mol	(69)
GPC	HPSEC/HPGPC-RID	Fast, convenient for detection, high resolution and good reproducibility, homogeneity and molecular weight can be detected simultaneously	*Bletilla striata* polysaccharide (pFSP) Mw 9.1 × 10^4^ Da	(4)
	HPSEC/HPGPC-ELSD		*Lepidium meyenii* polysaccharide (MP1) Mw 4.67 × 10^5^ Da	(70)
Combined techniques	HPSEC/HPGPC-MALLS	Less affected by sample structure or relative molecular weight, not necessary to use reference materials for calibration, high accuracy, can provide samples conformational information in solution	*Ligusticum chuanxiong hort* polysaccharide (LCP70-2A) Mw 6.46 × 10^4^	(71)

### Determination of Monosaccharide Composition

The combination of gas chromatography (GC) and mass spectrometry (MS) or flame ionization detection (FID) is usually used as a conventional analysis method for monosaccharide composition (72, 73). The first step in determining the composition of monosaccharides is to degrade polysaccharides into monosaccharides. The acid hydrolysis method is the most widely used, and its process varies depending on the type of polysaccharide. Next, due to the lack of volatility of carbohydrates, it is necessary to use sugar alcohol acetate, sugar acetonitrile acetate derivatives, methyl ether, trimethylsilyl oxime, trimethylsilyl ether, and other reagents to derivatize the hydrolysate, making it suitable for GC.

High performance liquid chromatography (HPLC) with ultraviolet detector (UVD) is becoming more commonly used for quantifying monosaccharides (74). Separation rate of monosaccharides can be accelerated accordingly in reversed-phase liquid chromatography with stationary phase of C18 (75). Polysaccharides are not retained on the C18 chromatographic column and do not contain chromophoric groups. Monosaccharides need to be derivatized so that the separation efficiency of chromatography and sensitivity of UVD can be improved to observable level. 1-phenyl-3-methyl-5-pyrazolone (PMP) is usually used as a probe molecule for its characteristic of quantitative reaction with carbohydrates under mild conditions (76). Besides, the monosaccharide composition of polysaccharides can be determined without derivatization by using sugar analysis column or amino column and equipped with RID or ELSD or pulsed amperometric detector (PAD).

High performance anion exchange chromatography-pulsed amperometric detector (HPAEC-PAD) is a technology used to analyze polysaccharide currently (77–79), as it can analyze the hydrolytic products without sample derivation (80). The mechanism of HPAEC-PAD method is to use the dissociation of mono or oligosaccharides by elution in medium with pH above 12. Then the anions are fully exchanged on an agglomerated shell type anion exchange resin column. The separated components go into PAD for detection. The composition of monosaccharides can be determined referring to standard samples (81).

**Figure 3:**
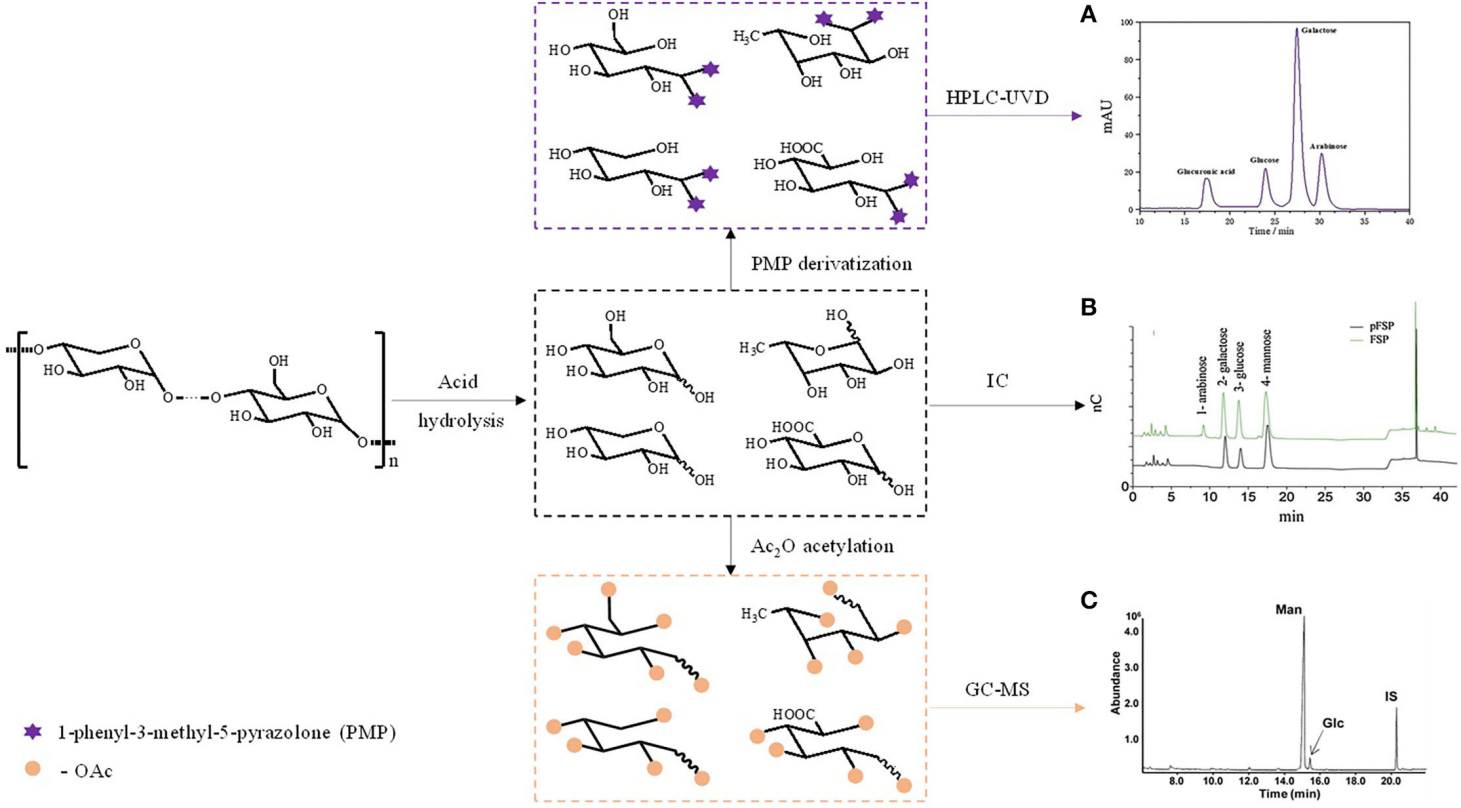
The general process of determining the composition of monosaccharides. **(A)** Monosaccharide composition of *Angelica sinensis* polysaccharide by HPLC-UVD; **(B)** Monosaccharide composition of polysaccharide from the fibrous roots and tuber of *Bletilla striata* by IC; **(C)** Monosaccharide composition of polysaccharide from *Dendrobium devonianum* by GC-MS.

Capillary electrophoresis can also be used to determine the monosaccharide composition of CHPs. Samples are labeled by reagents with acidic groups, and then assayed by high performance capillary electrophoresis (HPCE) and detected by laser induced fluorescence detector (LIFD) (82). The advantages and disadvantages of various methods are listed in Table 2. The general process of determining the composition of monosaccharides is shown in Figure 3.

**Table 2:** Comparison of determination methods of monosaccharide composition.

**Method**	**Characteristics**	**Application**	**References**
GC/MS	High sensitivity and accuracy, tedious derivatization process, lead to sample loss, cannot deal with acidic polysaccharides containing uronic acid, generate isomers	*Lycium barbarum* polysaccharide (LBP1) mannose: glucose: galactose: xylose: arabinose=6.52:78.12:8.85:1.81:4.69.	(83)
HPLC-PMP derivatization	Easy to operate, no isomers peaks, high specificity and accuracy, less sensitive than GC, low specificity of UVD, long analysis time, not suitable for ketose	*Dendrobium nobile Lindl* polysaccharide (DOP) D-glc*_*p*_*: D-man*_*p*_*=1.00:4.41.	(84)
Detecting directly by HPLC	Non-derivative, simplicity of operation, low sensitivity Sugar analysis columns: suitable for separating monosaccharides, separation conditions, convenient sample preparation, and a wide pH range of 1–14; Amino column: suitable for separating samples oligosaccharides, cheap, lifespan is short, with many precautions for use.		
HPCE	High sensitivity, unique effect in separating charged sugars, high requirements for instruments, complicated to operate, low reproducibility	*Acanthopanax senticosus* leaves polysaccharide (ASLP) Rhamnose: xylose: glucose: mannose: arabinose: galactose: glucuronic acid= 7.45:18.63:25.15:0.93:8.35:2.79:5.69	(85)
HPAEC-PAD	Not necessary for derivatization, high sensitivity, columns bearable for NaOH are needed.	The fibrous roots and tuber of *Bletilla striata* polysaccharide (pFSP) D-glucose: D-galactose: D-mannose=1:2.03: 3.45	(4)

### Glycosidic Bonds Identification

**Figure 4:**
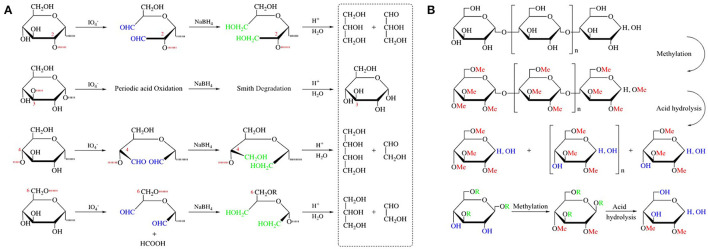
Chemical reactions related to the determination of glycosidic bonds. **(A)** Different polysaccharides are oxidized by HIO_4_, reduced by NaBH_4_, and then hydrolyzed with dilute acid to obtain different products; **(B)** Products obtained by methylation and acid hydrolysis of linear glucans and branched polysaccharides.

Chemical reaction is an important step in glycosidic bond identification methods, including acid hydrolysis, periodic acid oxidation, Smith cleavage, and methylation reactions (Figure 4). Partial acid hydrolysis is currently the most commonly used method (86), which can be used to detect the sequence of glycosidic linkages breaks and preliminarily infer the possible glycosidic bond types. By measuring the usage of periodic acid and the release of formic acid, the position of glycosidic bonds, the degree of polymerization of linear polysaccharides and the number of branches of branched polysaccharides can be judged. The characteristic of Smith's degradation is that only glycosidic bonds are broken by periodic acid, while the sugar residues that are not oxidized are still attached to the sugar chain. The key point to the methylation reaction is whether the methylation is complete (87). For polysaccharides containing uronic acid and aminohexose, whether methylated acid hydrolysis will cause side reactions. It is necessary to reduce the polysaccharide before methylation, if the samples contain uronic acid.

The common determination methods of glycosidic bonds in polysaccharides are shown in Table 3. And there are some new methods to identify the glycosidic bonds of polysaccharides. Amicucci described the development and application of a chemical method for producing oligosaccharides from polysaccharides. The released oligosaccharides are characterized by the advanced LC-MS methods with high sensitivity, accuracy, and throughput. The technique is first used to identify polysaccharides by oligosaccharide fingerprinting. The Fenton's initiation toward defined oligosaccharide groups (FITDOG) process was developed to produce oligosaccharides from polysaccharides. The process was initiated by a reaction between a metal catalyst, Fe^3+^, and an oxidizing agent, hydrogen peroxide, to produce reactive radical species that cleave glycosidic bonds. The radicals induce oxidative cleavage of the polysaccharide backbone to produce oligosaccharides that are representative of the parent polysaccharide structure (96).

**Table 3:** Common determination methods of glycosidic bonds of polysaccharides.

**Method**	**Information**	**Characteristic**	**References**
Partial acid hydrolysis	Ranking of stability of various sugar: the pyran sugar residue>the furan sugar residue, the hexose sugar>the pentose sugar, the sugar residues in main chain>the branched sugar residues	Poor selectivity, requires precise reaction conditions, not suitable for the complex mixed polysaccharides or heterogeneous structure products	(88–90)
Periodic acid oxidation	The position of glycosidic bonds; the degree of polymerization of linear polysaccharides; the number of branches of branched polysaccharides.	Carried out in the dark in an aqueous solution with pH 3–5, less polysaccharide samples are required	(4)
Smith degradation	Degradation product erythrose: 1 → 4 combined glycosidic bonds; glycerol:1 → 6, 1 → 2 glycosidic bonds or a reducing terminal glucose residue; monosaccharides such as glucose, galactose, mannose: 1 → 3 glycosidic bonds.	Combined with periodic acid oxidation	(91)
Methylation reaction	GC/MS spectral library; the standard PMAA spectrum; the ionization law of PMAA.	*α/β* stereochemical information cannot be obtained	(92, 93)
Enzymatic digestion	α-glucosidase hydrolysis α-glycosidic bond; β-glucosidase hydrolysis β-glycosidic bond.	Specific, few by-products, little digestive enzymes for polysaccharide	(94, 95)

### Spectroscopy Techniques

IR is mainly used to identify various functional groups, determine the α/β configuration of glycosidic bonds, distinguish five-carbon and six-carbon sugars, and identify different monosaccharides in polysaccharides (Table 4) (97, 98). The characteristic absorption peaks of polysaccharides are at 3,600–3,200, 3,000–2,800, and 1,200–1,000 cm^−1^ (99). Among them, polysaccharides contain lots of hydroxyl groups, which usually has a distinct broad stretching vibration absorption peak at the wavelength of 3,500–3,000 cm^−1^. The C-H stretching vibration can form a weak peak near 2,935 cm^−1^ (100). The absorption peak of sugar ring C-O-H, C-O-C is 1,000–1,100 cm^−1^. In addition, according to the absorption peak in the 1,170–700 cm^−1^ interval of the infrared spectrum, the size of sugar ring and the configuration of the glycosidic bond can be judged. Pyrannoside has 3 strong absorption peaks in the interval of 1,100–1,010 cm^−1^, while furanoside only has 2 absorption peaks in the corresponding region. The vibrations of the pyranose ring are at 917 and 770 cm^−1^, while the furanose ring is at 924 and 799 cm^−1^. Generally, 890 cm^−1^ is the characteristic peak of β-pyranoside bonds, and 840 cm^−1^ is the characteristic peak of α-pyranoside bonds.

**Table 4:** The IR wavenumbers of functional groups in polysaccharides.

**Functional group**	**Vibration mode**	**Wavenumbers/cm^**−1**^**
-OH	O-H, ν (stretching vibration)	3,700–3,100
	O-H, δ (scissoring vibration)	1,075–1,010; 1,120–1,105
-COOH	C=O	1,740–1,680
	C-O	1,440–1,395
	O-H	1,320–1,210
-O-COR	C=O	1,749–1,725
	C-O	1,245
-NH_2_	N-H, ν	3,450–3,380
	N-H, δ	1,650–1,500
=NH	N-H, ν	3,460–3,420
-NH+ 3	N=H	3,350–3,150
	N-H	1,650–1,550
-C-O-C	C-O (fatty ether)	1,150–1,060
	C-O (cyclic ether)	1,150–1,080
-CH_2_	C-H, ν	2,926–2,853
	C-H, δ	1,465
-CH_3_	C-H, ν	2,962; 2,872
-C=O -CHO	C=O	1,780–1,540
	C=O	1,740, 1,650
*α-*D-Glc*_*p*_*	C-H	855–833
β-D-Glc*_*p*_*		905–876
α-D-Gal*_*p*_*		839–810
β-D-Gal*_*p*_*		914–886
α-D-Man*_*p*_*		843–818
β-D-Man*_*p*_*		898–888
α-D-Xyl*_*p*_*		760–740
β-D-Arb*_*p*_*		855–830

Nuclear magnetic resonance spectroscopy (NMR) provides information about the structure of polysaccharides, which involves the identification of monosaccharides, α or β anomeric confirmation, glycosidic bonds type, and the sequence of repeating units in the polysaccharide chain. In the ^1^H-NMR spectrum, the chemical shifts of the polysaccharide signal are mainly distributed in δ 3.5–4.4 and δ 4.4–5.8 (anomeric proton region). Among them, the anomeric proton region plays a significant part of the structure analysis of polysaccharide, such as providing information on the configuration of polysaccharide residues. Generally, the chemical shift δ of the anomeric hydrogen of the pyranose residue with α configuration is larger than 5.0. For β configuration, the δ is less than 5.0. The configuration of the anomeric hydrogen can also be judged by combining the coupling constant *J*_1,2_ of the anomeric hydrogen and the vicinal hydrogen. For the β configuration, *J*_1,2_ = 7–9 Hz, and for the α configuration, *J*_1,2_ = 2–4 Hz. Unsaturated double bond in non-reducing end H4 falls in 5.9–6.0 ppm, H1 in 4.8–5.5 ppm and H2–H6 in 4.0–4.8 ppm. There are also some special proton signals that are helpful to determine the type of monosaccharide residues, such as δ 1.1–1.3, which may be the hydrogen at C6 position of fucose. When methyl (around 1.0 ppm), acetyl (around 2.0 ppm), sulfate and phosphate groups were substituted, the ^1^H chemical shift often shifted downward by about 0.2–0.5 ppm.

The ^13^C-NMR spectrum can confirm various carbon nuclei and distinguish the configuration and conformation of molecules in polysaccharide structure analysis. It can also be used to determine the substitution position and branch point of polysaccharide residues. In the ^13^C-NMR spectrum, the characteristic peaks of polysaccharides fall in 60–110 ppm. Among them, monosaccharide composition and sugar ring configuration can be determined by the isomeric carbon signal of 95–105 ppm. Allocarbons above 101 ppm can be distinguished as β-configuration, and between 95 to 103 as α-configuration. For C2–C5, 78–85 ppm is the carbon signal at the substituted position and 65–80 ppm is the one at unsubstituted position. Signals around 61 ppm belong to unsubstituted C6, and the substituted C6 signal moves to lower around 69 ppm. The signals in 96–110 ppm are the peaks of terminal carbons of sugar, and the other signals are the peaks of non-terminal carbons. The signals of unsubstituted C2, C3, C4, and C5 are at 78–70 ppm, while the unsubstituted C6 signals are at 64–60 ppm. The chemical shift of methyl carbon is about 35–40 ppm (100). Chemical shift of polysaccharide 13C substituted by methyl, acetyl, sulfate, or phosphate group shifts downward 6–7 ppm. Two-dimensional NMR (2D NMR) plays an indispensable role in the full attribution of ^13^C-NMR spectra of polysaccharides (101). Currently, NMR is a tool that can be used to analyze molecular structure completely independently, but it also has the problems of low sensitivity. Additionally, the severe overlap of the peaks makes the elucidation extremely complex, especially for Chinese herbal heteropolysaccharides.

The LC-MS methods have been developed and have brought remarkable advances in terms of sensitivity and specificity to the general analysis of polysaccharides, which can provide reliable molecular weights, and the information of fragments (102, 103). Xu et al. have developed a comprehensive method for quantitation of both neutral and acidic monosaccharides using ultra-high performance liquid chromatography triple quadrupole mass spectrometry (UHPLC/QqQ-MS) in dynamic multiple reaction monitoring (dMRM) mode. This method can achieve the separation, detection, and quantification of 14 PMP-derived monosaccharides (including fructose) and two sialic acids with label-free within 10 min, which has high sensitivity and wide linear range (104). Amicucci et al. presented a general LC-MS-based workflow for the *de novo* characterization of structurally diverse polysaccharides. This report presented the characterization of the maize polysaccharide by employing new analytical strategies, which is quantifying monosaccharide and glycosidic linkages by combining chemical depolymerization and UHPLC/QqQ-MS analysis. Partial acid hydrolysis paired with nano-HPLC/QTOF MS was used to analyze oligosaccharides sequencing. The elucidation of this complicated structure illustrates the high sensitivity, good reproducibility and fast speed of the analytical methods, which may serve as a general platform for polysaccharide analysis in the future (105).

## Advanced Structure Analysis

The traditional methods of studying the high-level structure of polysaccharides have limitations in application. A new technique as X-ray diffraction (XRD) method can obtain various information such as bond angle, bond length, configuration angle at the same time (106). But this technology requires that the polysaccharide sample must have high purity and crystallization. In other words, amorphous polysaccharides are not applicable. Except for polymers with triple helical structure, there is no other report using XRD to study chain conformation (57). Additionally, the Congo red experiment does not require special equipment and is easy to popularize. However, this method can only be used to determine whether the sample has a triple helix structure. It cannot elucidate the precise structure and has low sensitivity.

### Analytical Methods Based on the Theory of Polymer Dilute Solution

At present, the most commonly used analysis methods of advanced structure are HPLC combined with dynamic light scattering (DLS), static light scattering (SLS) or viscosity determination based on the theory of polymer dilute solution (107, 108). Normally, four main characteristics are considered when identifying polymer conformations, which concludes persistence length (q), molar mass per unit contour length (M_L_), diameter of chain (d), and contour length of chain (L). These four key parameters can be calculated by thermodynamic and hydrodynamic properties parameters including molecular weight (M_w_), radius of gyration (R_g_), intrinsic viscosity (η), diffusion coefficient (D), and hydrodynamic radius (R_h_), which can be obtained through the Kratky-Porod model, helical worm-like chain model, or models derived from them (109).

The HPLC-SLS technology is a simple but effective analysis method for advanced structure of polysaccharides (110). It is based on the light scattering properties of polymer solutions and the related characteristics of molecular mass, size and concentration. Various structural information such as M_w_, molecular mass distribution (MWD), and R_g_ can be obtained through formula *(1)* (111). Majority of studies still use Zimm mapping for data processing (112), while the Debye method can not only ensure accuracy but also shorten the experimental period (113). The exponent *v* of formula *(2)* is the slope of the linear regression of R_g_ to M_w_, which is one of the parameters that characterize the conformation of the polymer. Similar to *v*, the fractal dimension d_f_ is also a parameter that characterizes the tightness of the internal structure of the polymer, and it is calculated with formula *(3)*.

Because of the molecular thermal motion or Brownian motion in polymer solution, the phase of scattered particles in solution changes with time. The basic principle of DLS is that the frequency and the intensity of scattered light change and fluctuate with time. The values of R_h_ and D can be determined by DLS. R_h_ is an important parameter describing the size of a polymer in solution, and R_g_ is the root mean square value of the distance between the center of mass of the polymer and the axis of rotation. The form and rigidity of the polymer in a dilute solution can also be described by ρ (R_g_/R_h_) (114).

Viscometry is another simple method for measuring. The exponent α is a characteristic parameter of the high-level structure of polymers, which can be calculated according to formula *(9)*. And then the intrinsic viscosity η can be obtained by extrapolation using a viscometer according to Huggins equation *(10)* and Kraemer equation *(11)*. A large number of studies have combined these three methods for the analysis of the advanced structure of polysaccharides. Li S. et al. established an analytical method combining HPLC with multiple detectors to realize the analysis of β-Online separation and real-time detection of dextran aggregates and non-aggregates (115). The parameters, calculation formulas and the chain conformation information reflected by them are listed in Table 5.

**Table 5:** The parameters, calculation formulas, and the chain conformation information reflected by them.

**Formula**	**Diameter**	**Range**	**Conformation**	**References**
*(1)* $\frac{K_{c}}{R_{\theta }}=\frac{1}{M_{w}}\left[1+\frac{16\pi^{2}n^{2}}{3\lambda^{2}}R_{g}^{2}\text{si}{\text{n}}^{2}\left(\frac{\theta }{2}\right)\right]+2A_{2}c$	*v*	0.2–0.4	A tightly coiled conformation with high branches	(116, 117)
		0.5–0.6	A compliant molecule	
*(2)* $R_{g}=KM_{w}^{v}$		0.6–1.0	A rigid or semi-rigid rod	
*(3)* *d*_*f*_ = 1/*v*	d_f_	1.0	A rigid rod-like structure	(116, 118)
*(4)* $I_{s}\left(\omega \right)=I_{0}\left(\omega_{0}\right)\frac{C\Gamma }{\left(\omega -\omega_{0}^{2}\right)+\Gamma^{2}}$		5/3–2	A linear polymer with a Gaussian coil shape	
		2.5	A branched structure	
*(5)* *G*^(2)^(*q*, τ) = *A*[1+β|*g*^(1)^(*q*, τ)^2^|]		3	A compact and uniform spherical structure	
*(6)* $|g^{\left(1\right)}\left(q,\tau \right)|=\overset{\infty }{\underset{0}{\int }}G\left(\Gamma \right)d\Gamma$	ρ	0.77	A hard sphere conformation	(119)
*(7)* Γ = *Dq*^2^		1.0–1.1	A highly branched conformation	
*(8)* $R_{h}=\frac{k_{B}T}{6\pi \eta_{0}D}$		1.5–1.8	A compliant molecule	
		> 2	A worm-like or rigid structure	
*(9)* $\left[\eta \right]=KM_{w}^{\alpha }$	α	0.5	A spherical structure	(120, 121)
*(10)* $\eta_{sp}/C=\left[\eta \right]+K^{\prime }{\left[\eta \right]}^{2}C$		0.6–0.8	A elastic random curl conformation	
*(11)* $\left(In\eta_{r}\right)/C=\left[\eta \right]+K^{\prime \prime }{\left[\eta \right]}^{2}C$		>0.8/> 1.0	A rigid chain conformation	

### Microscope Observation Methods

High resolution and large depth of field are two main features of scanning electron microscope (SEM). The rough surface with certain fluctuations of polysaccharides can be directly observed by it (122). The surface morphology of polysaccharides significantly differs from each other due to various sources of polysaccharides. Spherical, flake and branched structures have been observed before, among which, spherical or flake morphology are most common (123, 124). And the extraction, purification, and preparation conditions could also influence on the surface morphology of polysaccharides (125). For example, three polysaccharides from *Sagittaria sagittifolia* L. separately obtained with hot water, ultrasound-assisted, and subcritical water extraction presented different SEM images (21). The different surface morphology of polysaccharides acquired by SEM can preliminarily infer the force between molecules. Polysaccharides usually have honeycomb-like structures, rough surfaces, and porous structures, indicating that the interaction among molecules is weak (126). A flake layer with unregular curls was observed indicating the strong attractions between functional groups on the surface to create the polysaccharide chains aggregation (127). The variation on morphology and shape mainly contributed to the changes of intramolecular hydrogen bonds (127). And the morphology and shape will affect the physical and chemical properties of polysaccharides. For instance, the smooth surface of polysaccharide probably had negative effect on the rehydration performance to reduce the solubility of polysaccharide itself (4).

Atomic force microscopy (AFM) is an analytical instrument that can study the three-dimensional (3D) surface morphology of polymers on the nanoscale in air and liquid (128). In AFM, when the probe scans the surface of the sample with a constant force between the probe and the sample, its motion trajectory can be recorded and converted into an integral 3D image. AFM does not require any special treatment on the sample, such as copper or carbon plating, which will cause irreversible damage to the sample. The disadvantage of AFM is that the imaging range is too small, the speed is slow, and it is too much affected by the probe. In early research, AFM was usually used to calculate the shape of the chain, including the diameter of the chain, the length of the chain, and the chain distribution of the polymer (129, 130). Zhao et al. used an AFM to observe the aggregation state, morphology and size of *Schisandra* polysaccharide particles in pure water. The heights of the particle were in the range of 1–5 nm and found that the polysaccharide particles had agglomeration phenomenon at 100 °C (131). The agglomerations suggest polysaccharide molecules have gathered and that their structures were branched and entangled (132).

### Other Methods

According to the different characteristics of the sample's absorption of left-handed and right-handed circularly polarized light, circular dichroism (CD) can be used to observe the structure of polysaccharides (133, 134). Zhang et al. analyzed the CD spectra of the *Cistanche deserticola* polysaccharide in the range of 190–300 nm. They found that some fragments of the *Cistanche deserticola* polysaccharide mainly exist in an ordered structure, and locally form a spiral structure (135). The results of CD analysis of the *Artemisia sphaeroides* polysaccharide before and after the sulfonation showed that the shielding effect of the charged components promoted the transformation of the polysaccharide chain to a more rigid conformation (136).

The differential scanning calorimetry (DSC) can not only describe the change about temperature between the sample and the reference but also record the change law between the heat difference and the temperature in real time. Liu et al. used this method to study the conformational transition process of *Ganoderma lucidum* polysaccharide 20 (GLP20). As a result, in the DSC heating curve, the endothermic peak indicated that the intermolecular hydrogen bond was broken, and GLP20 changed from a triple helical structure to a single-stranded random coil (137).

Polysaccharide macromolecules are usually in a dynamic equilibrium state of multiple spatial conformations, and the structural information provided by NMR is the average information of these dynamic conformations. A series of spatial structures that meet the NMR data and truly reflect the dynamic equilibrium of the sugar chain are obtained through the method of molecular calculation. Usually, molecular dynamics simulation (MDS) can depict the chain conformation of macromolecules with more than 10 repeating units. Based on the lowest energy state, the optimal conformation of the polysaccharide could be confirmed after simulated annealing (57). For instance, the complex 3D conformation of lentinan that can retain its triple-helix through hydrogen network was revealed using MDS and rigid macromolecule docking, as well as spectral methods (40).

## Structural Modification

**Figure 5:**
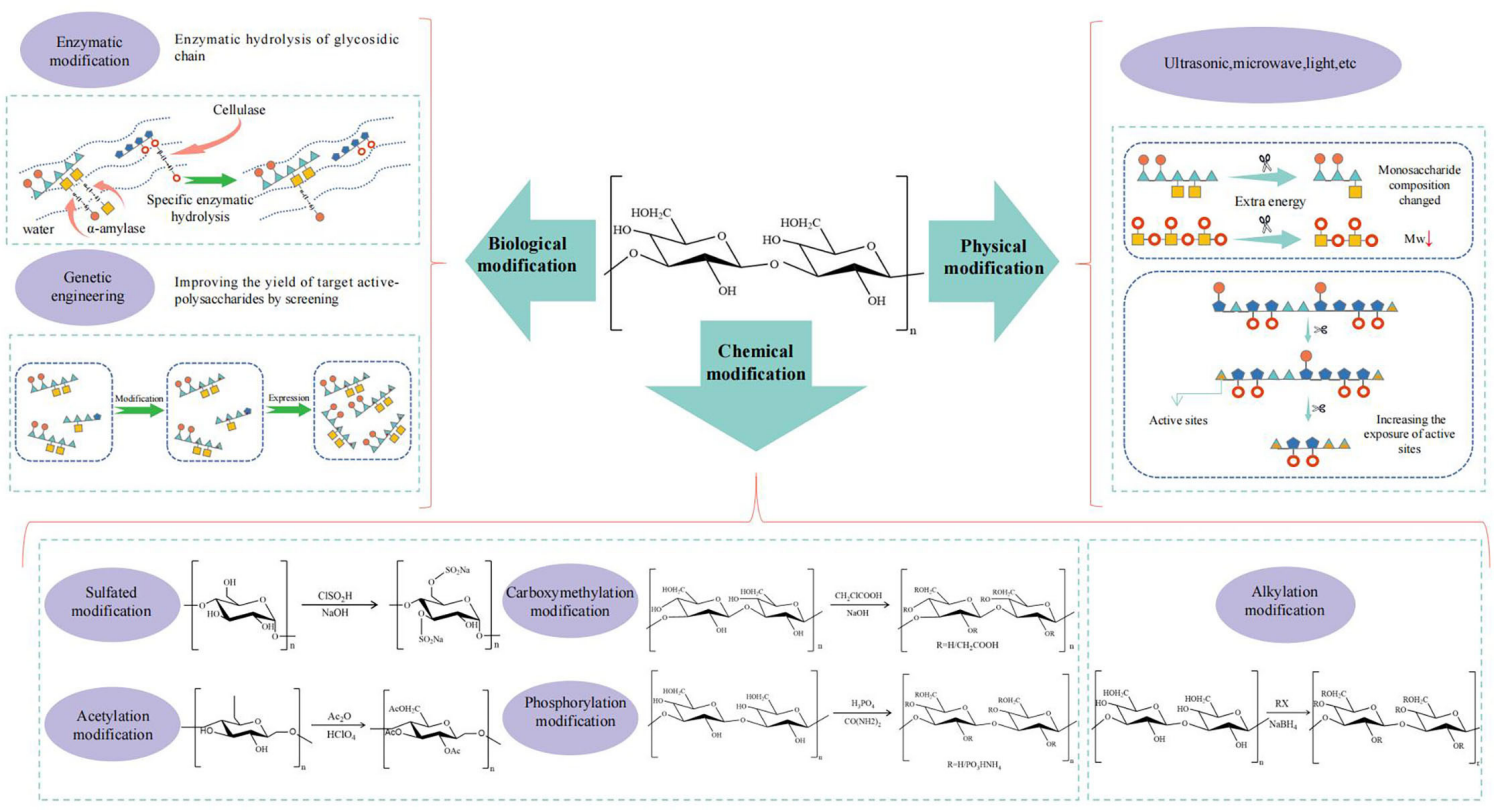
The schematic diagram of relationship between structural modification and biological activity of CHPs.

The common methods of structural modification in CHPs are mainly divided into three categories, namely chemical modification, physical modification, and biological modification (Figure 5).

### Chemical Modification

Chemical modification is the most common approach to tailor polysaccharide structure by inletting the required groups and thereby changing its original activity as well as producing new bioactivities (138). For instance, it was reported the anti-tumor activity of *Poria cocos* polysaccharide after modifications exhibit the stronger than the former (139). The modification was performed to achieve good water solubility, high chain rigidity and moderate molecular weight, which may be the reason for the improvement of its anti-tumor activity.

The branched chains of the polysaccharide can be modified by sulfation, phosphorylation, carboxymethylation, acetylation, etc., to enhance the bio-activity.

In sulfated modification, sulfuric acid groups are used to replace the hydroxyl, carboxyl, and amino ending on the sugar chain to exert activities. The degree of sulfation is positively related to anti-HIV and anticoagulant activity of CHPs. Firstly, sulfate groups are considered to be one of the determinants of anti-HIV activity of CHPs (8, 140). Then, sulfate groups can enhance the anticoagulant activity of CHPs by generating the high level of negative charge density. The sulfated polysaccharides obtained from *Zingiber officinale* with the higher degree of sulfation and suitable molecular weight showed better anticoagulant activities (141). In addition, CHPs exert their antioxidant activity by providing hydrogen atoms from polysaccharide chain and the presence of sulfated groups could increase the ability to provide hydrogen atoms (142, 143). In summary, sulfated polysaccharide are increasingly causing more attention, as they have been proved to improve structural properties and promote a variety of bioactivity.

The water solubility of polysaccharide can be improved by carboxymethylation with introduction of carboxymethyl into polysaccharide chain and thereby enhancing the biological activities of unmodified polysaccharides. After carboxymethylation, the molecular weight of polysaccharide from *Cyclocarya paliurus* decreased and correspondingly the molar ratio of monosaccharide composition changed (144). Among carboxymethylated polysaccharides, the antioxidant activity aims to terminate free radicals against oxidation reactions from occurring by increasing the ability of chelating transition metal ions and providing single electron or hydrogen atoms with the increased content of -COOH (145). It is worth mentioning that there is a positive correlation between antioxidant activity and the degree of carboxymethylation within a certain range (146). In addition, carboxymethylation can also enhance other biological activities of CHPs, such as anti-tumor and immune regulation activity (147). However, the mechanisms of action of carboxymethylated polysaccharides are still unclear and need to be explored forward.

On top of carboxymethylation, acetylation with the hydroxyl oxygen or amino nitrogen as the acetyl substitution sites can also enhance the water solubility of CHPs, mainly due to the exposure of hydroxyl groups from polysaccharide caused by the extension of polysaccharide chains (148). After the exposure of hydroxyl, the supply of hydrogen could be raised up, thereby enhancing the antioxidant activity of CHPs (149). It was found that the acetylated pumpkin polysaccharides with high degree of substitution had better antioxidant activity than those with low degree of substitution, and all of them perform better than those without modification (150). In addition, the potential application of acetylated CHPs is a feasible strategy to be an immunotherapeutic adjuvant (151).

A specific method used to modify the main chain of polysaccharides is the alkylation reaction by alkyl and long-chain aromatic alcohol for improving water solubility, reducing the viscosity of the solution, and thus improving the bioavailability. A good example was that *Ganoderma lucidum* polysaccharide was hydroxypropylated to improve the aqueous solubility and its antioxidant activity was significantly enhanced (152). An acceptable reason is that the hydroxyl groups in the molecules are more easily exposed to the reactive oxygen species (ROS), including free radicals and oxidants.

However, it should be noted that the appropriated degree of substitution is crucial to the activity. The crude polysaccharides of *Cordyceps militaris* were successfully modified by carboxymethylation and acetylation, but the expressed α-glucosidase inhibitory activity did not change significantly (153). To sum up, the change of the structure in polysaccharides has certain impacts on the biological activity with the degree of substitution and the substituent position as important factors affecting the result.

### Physical Modification

In the physical modification, the degradation products with low molecular weight were obtained by serials of ultrasound, high energy radiation, microwave, and light to cut off some chemical bonds in the main chain due to high molecular weight of polysaccharides showing characteristics of high viscosity and poor solubility against absorption and utilization *in vivo* (154).

Ultrasonic treatment is the most commonly used method in physical modification. The ultrasonic treatment in the process of polysaccharide can break glycosidic bond to reduce the molecular weight, the viscosity, and enhance the solubility (155). It has been reported that the decreased molecular weight is positively correlated with ultrasonic intensity (154). In the process of ultrasonic, monosaccharide composition and uronic acid content would be also monitored as important indexes (156). The molecular weight of corn whisker polysaccharides after ultrasonic degradation decreased significantly, but the monosaccharide composition did not change. Interestingly, the molar ratio of mannose to galactose decreased significantly, suggesting that mannose and galactose residues may be the main active sites in the process of ultrasonic degradation. In the process, ultrasonic treatment also exposed more groups such as uronic acid, and thus provided more binding sites for increasing of α-glucosidase inhibitory activity (157).

### Biological Modification

The biological modification for polysaccharides mainly involves enzymatic modification and genetic engineering. In enzymatic modification, specific biological enzymes are often used to degrade polysaccharides, and in which the non-reducing ends are cleaved resulting in double bonds forming. The molecular weight and solution viscosity of polysaccharides are decreased after side-chain detachment occurring by enzyme-modified treatment beyond the change of monosaccharide composition and biological activities enhancing (125, 158). It was found that the antioxidant activity of *Morus alba* L. polysaccharides hydrolyzed by cellulase was higher than original polysaccharides, which may be attributed to the exposure of more active groups of polysaccharides (159). After enzymatic hydrolysis, the primary structure of polysaccharide from *Hericium erinaceus* with the shorter branched chains did not change, which led to the decrease of molecular weight, the increase of glucose content and the enhancement of immunomodulatory activity of the polysaccharide (160). At present, it should be noted that most of the enzymes can be used in enzymatic modification as degradable enzymes, and the applicable types are limited, which blocks the usage of this method.

To promote the industrial production of polysaccharides, genetic engineering is employed to control the biosynthesis pathway of polysaccharides by manipulating the gene expression of microorganisms or introducing exogenous genes in microorganisms for obtaining target active-polysaccharides (161). The proportion of galactose and mannose in polysaccharides and the antioxidant activity of polysaccharides were greatly boosted by efficient expression of the Vitreoscilla hemoglobin gene in Ganoderma lucidum (162). However, genetic engineering is difficult to be operated and foreign genes are not easy to obtain, which still needs to be further developed.

## Conclusions and Prospects

Up to now, researches on CHPs are still in the initial stage with many shortcomings. It can be concluded that there has been a conventional structure analysis procedure and common structure-modified approach, but no unified conclusion on the structure-activity relationship of CHPs.

The biological activities of CHPs are closely related to its structure. Firstly, according to the facts we consolidated, the activity of polysaccharide performs the best only in the relative optimum molecular weight range while the determination of the specific range still needs more work. Secondly, the type and proportion of monosaccharide composition has a certain influence on the activity of polysaccharides. Nevertheless, the rules reflected different polysaccharides on activities are multifarious. Thirdly, as for the effect of glycosidic bonds on the activity of CHPs, CHPs with anti-tumor effect composed of the β-(1 → 3)-*D*-glucan as the main chain and the β-(1 → 6)-*D*-glucan randomly as the branched chain were in-depth studied. However, the studies on the glycosidic bonds of CHPs with other activities are not systematic enough, and most of them are in a state of scattered research. Fourthly, the DB of CHPs affects biological activity by changing molecular weight and conformation, and an ideal DB value may exist. This might be due to the fact that a high DB value reduces water solubility, whereas a low DB value results in fewer binding sites. The attention should be paid to the study of the type and length of branches, so as to reveal the mechanism of their influence on biological activity. Finally, the polysaccharides of triple helix conformation show excellent activities. On the one hand, the study of mechanism between triple helix conformation and activities should be strengthened. On the other hand, the research of CHPs with other conformations should not be ignored.

With regard to molecular modification, chemical modifications are much more focused due to obviously enhancing the activity of CHPs by inletting the required groups, wherein degree of substitution and position of substituent play the important role. In addition, physical and biological modification could affect the activity of polysaccharides by changing their molecular weight, monosaccharide composition, and physicochemical properties. Physical modification can degrade the main chain of polysaccharides, which is relatively friendly to the acquisition for small molecule-weight of polysaccharides, but the method is too unstable. In addition, enzymes are widely used in biological modification with high efficiency and controllability, but the categories and amounts of available enzymes are finite. Finally, genetic engineering technology can achieve a large number of targeted polysaccharides, but this method is difficult to operate and exogenous genes are not easy to obtain.

In light of the aforementioned issues, some modern equipment also promoted the development of structural analysis techniques for polysaccharide. Besides, multidisciplinary approaches can help researchers think outside the box. MDS and other calculation techniques can better examine the three-dimensional structure of polysaccharides. Through the combined use of multiple technologies, interdisciplinary methods are applicable to the structural characterization, especially the analysis of chain conformation of CHPs. Meanwhile, combined with the structural modification, the desired biological activity could be obtained by degrading or introducing the target groups. Then, in wake of structural elucidation, some rules are deduced among structure-activity relationship of polysaccharide, which may speed up the application of CHPs.

## Author Contributions

BW and LY: investigation and writing original draft. SG, LW, and MY: writing. LF and XJ: reviewing. All authors contributed to the article and approved the submitted version.

## Funding

This work was financially supported by the National Key research and development program of China (2018YFC1706906), Double First-Class University project of China Pharmaceutical University (CPU2018GF07 and CPU2018PZQ19), National Natural Science Foundation Committee of P.R. China (Nos. 81703775 and 81973536), and the Special Fund for Transformation of Scientific and Technological Achievements in Jiangsu Province (BA2020077) and the Project Program of State Key Laboratory of Natural Medicines, China Pharmaceutical University (SKLNMZZ202025).

## Conflict of Interest

The authors declare that the research was conducted in the absence of any commercial or financial relationships that could be construed as a potential conflict of interest.

## Publisher's Note

All claims expressed in this article are solely those of the authors and do not necessarily represent those of their affiliated organizations, or those of the publisher, the editors and the reviewers. Any product that may be evaluated in this article, or claim that may be made by its manufacturer, is not guaranteed or endorsed by the publisher.
